# The breadth of maternal HIV-1 specific neutralizing antibodies is not associated with a lower risk of mother-to-infant transmission

**DOI:** 10.1186/1742-4690-9-S2-P44

**Published:** 2012-09-13

**Authors:** A Chaillon, T Wack, M Braibant, L Mandelbrot, S Blanche, J Warszawski, F Barin

**Affiliations:** 1 INSERM U966 Research Unit, F. Rabelais University, Tours, France; 2 INSERM U1018, Le Kremlin-Bicêtre, Paris, France; 3 Louis Mourier Hospital, Paris-Diderot University, Paris, France; 4 Necker Hospital, EA 3620, Paris-Descartes University, Paris, France; 5 Inserm U1018, Le Kremlin-Bicêtre, Paris, France

## Background

It has been hypothesized that neutralizing antibodies (nAbs) should have broad specificity to be effective in protection against diverse HIV-1 variants. The mother-to-child transmission of HIV-1 is a model that provides the opportunity to examine whether the breadth of maternal nAbs would be associated with protection of infants from infection.

## Methods

Samples were obtained at delivery from 57 transmitting mothers (T) matched with 57 non-transmitting mothers (NT) enrolled in the multicenter French Perinatal Cohort (ANRS EPF CO1) between 1990 and 1996. The mothers did not receive antiretroviral therapy during pregnancy, and did not breastfeed their infants. Sixty-eight (59.6%) and 46 (40.4%) women were infected by B and non-B viruses, respectively. Neutralization assays were carried out in TZM-bl cells using a panel of 10 primary isolates of 6 clades (A, B, C, F, CRF01_AE, CRF02_AG) selected for their moderate (tier 2) or low (tier 3) sensitivity to neutralization. The presence and titers of Nab to each strain, and the breadth of maternal nAbs at delivery were compared between T and NT mothers.

## Results

Although there was a trend for both higher frequency and higher titers of nAbs in NT mothers vs T mothers for almost all the primary isolates that were tested, the differences were not statistically different when considering the entire population. However a few statistically significant differences were observed with higher frequency or higher titers of nAbs toward several individual strains in NT mothers when analyzing separately the B-infected or non-B infected mothers.

## Conclusion

Our study confirms that the breadth of maternal nAbs is not associated with protection of infants from infection. However it suggests that, depending of the population, some primary isolates could be indicators of nAbs associated with a lower risk of MTCT.

